# Bridge Deformation Monitoring Combining 3D Laser Scanning with Multi-Scale Algorithms

**DOI:** 10.3390/s25133869

**Published:** 2025-06-21

**Authors:** Dongmei Tan, Wenjie Li, Yu Tao, Baifeng Ji

**Affiliations:** 1 Sanya Science and Education Innovation Park, Wuhan University of Technology, Sanya 572000, China; jbfeng@whut.edu.cn; 2 School of Civil Engineering and Architecture, Wuhan University of Technology, Wuhan 430070, China; 348107@whut.edu.cn (W.L.); taoyu123456@whut.edu.cn (Y.T.)

**Keywords:** bridge structure, deformation monitoring, three-dimensional laser scanning, point cloud processing, least-squares plane fitting, Multi-Scale Model-to-Model Cloud Comparison (M3C2) algorithm

## Abstract

To address the inefficiencies and limited spatial resolution of traditional single-point monitoring techniques, this study proposes a multi-scale analysis method that integrates the Multi-Scale Model-to-Model Cloud Comparison (M3C2) algorithm with least-squares plane fitting. This approach employs the M3C2 algorithm for qualitative full-field deformation detection and utilizes least-squares plane fitting for quantitative feature extraction. When applied to the approach span of a cross-river bridge in Hubei Province, China, this method leverages dense point clouds (greater than 500 points per square meter) acquired using a Leica RTC360 scanner. Data preprocessing incorporates curvature-adaptive cascade denoising, achieving over 98% noise removal while retaining more than 95% of structural features, along with octree-based simplification. By extracting multi-level slice features from bridge decks and piers, this method enables the simultaneous analysis of global trends and local deformations. The results revealed significant deformation, with an average settlement of 8.2 mm in the left deck area. The bridge deck exhibited a deformation trend characterized by left and higher right in the vertical direction, while the bridge piers displayed noticeable tilting, particularly with the maximum offset of the rear pier columns reaching 182.2 mm, which exceeded the deformation of the front pier. The bridge deck’s micro-settlement error was ±1.2 mm, and the pier inclination error was ±2.8 mm, meeting the Chinese Highway Bridge Maintenance Code (JTG H11-2004) and the American Association of State Highway and Transportation Officials (AASHTO) standards, and the multi-scale algorithm achieved engineering-level accuracy. Utilizing point cloud densities >500 pt/m^2^, the M3C2 algorithm achieved a spatial resolution of 0.5 mm, enabling sub-millimeter full-field analysis for complex scenarios. This method significantly enhances bridge safety monitoring precision, enhances the precision of intelligent systems monitoring, and supports the development of targeted systems as pile foundation reinforcement efforts and as improvements to foundations.

## 1. Introduction

To ensure the proper use and safe operation of bridge structures throughout their service life, regular deformation monitoring is essential to gather deformation data under load conditions [1]. When deformation exceeds the structural load-bearing capacity, it can result in catastrophic consequences, jeopardizing the safety of lives and property. Therefore, establishing a long-term, high-precision deformation monitoring system is vital for ensuring the safe utilization of bridge structures and the smooth operation of transportation systems [2].

Traditional deformation monitoring technologies primarily rely on total stations, remote sensing (RS), global navigation satellite systems (GNSSs) [3], and interferometric synthetic-aperture radar (InSAR). At present, the Global Positioning System (GPS) serves as the foundation for measuring the displacement of long-span bridges. Nickitopoulou, Protopsalti, and Stiros [4] suggest that at the 98.5th percentile level, the standard accuracies for horizontal and vertical measurements can reach up to 15 mm and 35 mm, respectively, without serious errors such as circumferential jumps or multipaths. Casciati and Fuggini [5] confirmed the accuracy at the sub-centimeter level through static and dynamic tests. In the monitoring of crustal deformation on the Qinghai–Tibet Plateau conducted by Xingxing Li et al. [6], the BeiDou Navigation Satellite System (BDS)/GPS Precise Point Positioning (PPP) fusion method was adopted, and the horizontal accuracy reached 2.8 mm. Although integrating accelerometers and tilt sensors can partially improve accuracy, this induces a significant increase in the complexity of the system, thereby limiting its practical engineering applications [7].

The emergence of Terrestrial Laser Scanning (TLS) technology has introduced an innovative solution for deformation monitoring. With its non-contact operation, high efficiency (millions of points per second), and exceptional precision (at a sub-millimeter level), this technology has been widely adopted in the field. Compared to traditional monitoring techniques, TLS not only meets the accuracy requirements for deformation monitoring but also facilitates the rapid acquisition of deformation data and the construction of high-precision three-dimensional (3D) point cloud models. Utilizing this technology, millions of 3D points (i.e., point cloud data) can be collected in just a few minutes, providing a reliable data foundation for constructing comprehensive models [8].

TLS has demonstrated significant technical advantages in structural inspection and deformation assessment, providing a new paradigm for monitoring complex engineering structures [9,10,11]. Wen et al. [12] compared TLS with leveling instruments in bridge load tests and confirmed that TLS offers higher accuracy. Similarly, studies by Song-in et al. [13] and Xiao-ju et al. [14] further validated that TLS meets the precision requirements for in situ bridge deformation monitoring. Lubowiecka et al. [15] proposed an integrated approach that combines laser scanning, ground-penetrating radar, and finite element analysis and applied it to the assessment of medieval masonry bridges. Sedek and Serwa [16] demonstrated the significant potential of TLS in building defect detection, quality control, and spatial deformation monitoring. Park et al. [7] utilized TLS for monitoring the deformation of steel beams and extracted deformation information from single-point cloud data. Xu et al. [17,18] and Yang et al. [19] employed TLS to analyze the deformation behavior of composite arch structures under monotonic loading, focusing on the approximate surface model of the masonry arch bottom at the intrados of reinforced concrete supports. Additionally, Deng Y et al. [20] proposed an algorithm for automatically extracting arch axes from massive point cloud data and applied this algorithm to the 800-year-old Lugou Bridge in Beijing. The structural parameters of the bridge were identified based on the extracted arch axes. Collectively, these studies demonstrate the efficiency and feasibility of TLS in quality inspection and offer various evaluation methods for different structural objects. However, the processing of TLS point clouds still encounters challenges, including multi-temporal registration, noise suppression, and multi-scale deformation quantification, which hinder its widespread application in bridge health monitoring.

During the operational phase of bridges, deformation typically exhibits a regular distribution pattern, which can be extracted using multi-temporal point cloud registration techniques. Point cloud registration aligns point clouds from different coordinate systems into a unified reference frame through rotation, translation, and scaling. This process consists of two main stages: coarse registration and fine registration. Coarse registration provides initial values for fine registration, and its accuracy and efficiency directly influence the final results. Integrating geometric features and structural characteristics of the bridge into the registration process represents a core challenge in improving the accuracy of deformation extraction.

Within the field of coarse registration, Aiger [21] proposed the 4-Points Congruent Sets (4PCS) algorithm, which randomly selects a coplanar four-point base in the source point cloud and searches for approximately congruent coplanar four-point sets in the target point cloud. However, this algorithm tends to converge to local optima when the point cloud overlap is low, leading to registration failures. To address this limitation, Theiler et al. [22,23] developed the Keypoint-based 4-Points Congruent Sets (K-4PCS), which improves registration efficiency by incorporating keypoints. For fine registration, the Iterative Closest Point (ICP) registration algorithm serves as a classical 3D point cloud registration method that achieves high-precision alignment by minimizing the Euclidean distances between corresponding points. However, the ICP algorithm is not well suited for processing point cloud data that include non-overlapping regions. To address this limitation, Chetverikov et al. [24] introduced the trimmed ICP algorithm. This approach sorts distance residuals in ascending order and retains a subset of corresponding points based on the overlap ratio. As a result, it effectively facilitates the registration of partially overlapping point clouds, although it exhibits suboptimal performance when dealing with asymmetric deformations.

During 3D laser scanning, the generation of noise points is inevitable, which significantly affects subsequent data processing. Therefore, point cloud denoising is a critical preprocessing step. Based on the robust optimization of local geometric features, Narváez et al. [25] proposed Weighted Principal Component Analysis (WPCA), which enhances computational efficiency and robustness by assigning low weights to noise points. For the removal of statistical outliers, Rusu et al. [26] introduced a statistical filtering method that identifies noise through neighborhood distance analysis; however, the selection of thresholds depends on empirical parameters. Regarding fast clustering-based denoising, Zhou et al. [27] developed a dual-value-based non-iterative denoising method that overcomes the slow processing speed of traditional k-means and k-d tree approaches while maintaining high accuracy. Although denoising significantly enhances the quality of point clouds, the large volume of data still contains a considerable number of redundant points after processing, which poses challenges for subsequent analysis. Therefore, point cloud simplification is a crucial preprocessing step. Weir et al. [28] proposed voxel grid simplification for efficient compression through spatial gridding, although uniform sampling risks losing edge features. Sun et al. [29] improved this method by replacing all points within a voxel with the nearest point to the grid center, enhancing accuracy at the cost of increased computational complexity. Kim et al. [30] developed a curvature-based simplification approach that preserves structural details but introduces holes in flat regions. To address these issues, Qi et al. [31] formulated simplification as an optimization problem by constructing feature loss and density uniformity loss functions, effectively balancing global uniformity and feature retention through optimization algorithms.

Bridge deformation monitoring encompasses three core tasks: deck deflection analysis, pier verticality detection, and overall deformation assessment. Deck deflection monitoring involves quantifying displacements along the central axis and in the direction perpendicular to it, and it is often combined with pier verticality inspection. Overall deformation monitoring focuses on macro-scale trend analysis, typically employing methods such as Digital Elevation Model of Difference (DOD), Direct Cloud-to-Cloud Comparison (C2C), and Cloud-to-Model Distance (C2M). The DOD method identifies terrain changes by comparing multi-temporal Digital Elevation Model (DEM) data but remains limited to two-dimensional (2D) pixel-level analysis, struggling with 3D model comparisons, and thereby exhibiting significant errors in complex terrains [32,33]. The C2C method enables rapid change detection for dense point clouds without requiring data matching or meshing; however, its accuracy suffers from point cloud density, noise, and point spacing effects, making it suitable only for rough assessments. The C2M method relies on manually constructed reference grids or theoretical models, achieving high precision in flat terrain but introducing interpolation errors in data gaps while suffering from low computational efficiency [34,35]. To overcome these limitations, Lague et al. [36] proposed the Multi-Scale Model-to-Model Cloud Comparison (M3C2) algorithm, which directly detects changes under complex terrain conditions without meshing and demonstrates minimal sensitivity to point density, surface roughness, or sampling variations. This method has been successfully applied in lakeshore erosion monitoring, retaining wall displacement analysis, and offshore rockfall monitoring [37,38,39]. This study employs the M3C2 algorithm, which integrates bridge geometric features with load-response characteristics to facilitate multi-scale deformation extraction. This approach effectively addresses the spatial resolution and applicability limitations of traditional methods. However, current techniques still demonstrate considerable errors in low-overlap registration, face challenges in balancing noise filtering with feature retention, and lack a systematic analysis of the correlations between global and local deformations.

Bridge deflection, defined as vertical deformation along the central axis under loads or thermal effects, plays a crucial role in structural safety assessment. Traditional monitoring techniques (e.g., GNSSs, total stations, laser interferometry, deflectometers) remain widely used but face notable limitations: GNSSs require specialized equipment; total stations entail prolonged monitoring cycles; and laser interferometry and deflectometers rely on prisms or reflective targets, which limits real-time synchronous monitoring. These methods generally suffer from limitations associated with single-point measurements and fail to capture comprehensive full-field deformation information. Recently, TLS has emerged as a promising alternative for monitoring bridge deflection. Lichti et al. [40] were the first to apply TLS to timber bridge deflection monitoring, reconstructing cross-sections using second-order least-squares fitting across 95 static load tests (with a maximum load of 65.75 tons). They achieved root-mean-square error (RMSE) values of ±9.1 mm (bottom) and ±4.9 mm (top), which surpassed the results obtained from digital image correlation. Zogg and Ingersand [41] further validated the accuracy of TLS through static load tests on the Felsenau Viaduct, demonstrating a deviation of less than 3.5 mm from leveling measurements (with a mean residual of less than 1.0 mm) and confirming its applicability in engineering contexts. Lidu et al. [42] introduced a sliding-window deflection analysis method for continuous deformation curve extraction, whereas Xiaolong D et al. [43] combined target-based point clouds with finite element simulation to reduce detection errors by approximately 30%. Xu et al. [44] extended TLS applications to landslide monitoring and proposed fitting- and centroid-based methods that provide valuable references for complex structural deformation analysis.

These studies collectively demonstrate that TLS overcomes traditional limitations by utilizing high-density point clouds and non-contact measurements. However, challenges persist in data processing efficiency, deformation identification capabilities, noise suppression, and multi-temporal registration accuracy, highlighting the need for further optimization efforts.

In the aforementioned literature, for traditional monitoring techniques, the efficiency and accuracy of single-point monitoring are limited, and it is difficult to obtain the deformation information of the entire field. However, three-dimensional laser scanning technology also has problems that need to be solved urgently. Low-overlap point clouds can lead to large registration errors, and existing algorithms are sensitive to asymmetric deformations. The quantification on multi-scale deformation has not been sufficiently achieved in current research, and most research methods find it difficult to analyze global and local deformation simultaneously. Based on this, a multi-scale analysis method is proposed here that integrates the M3C2 algorithm and least-squares plane fitting. First, the ICP registration and M3C2 algorithms were adopted to detect global deformation of the bridge. Then, based on the least-squares fitting algorithm, a more refined local detection of the important deformation components of the bridge was performed. By introducing geometric constraints to optimize the registration parameters, the registration error for non-overlapping point clouds was controlled to within 0.5 mm. A curvature-adaptive cascade denoising algorithm was employed, achieving noise removal while preserving more than 95% the structural features. A multi-level cross-sectional analysis model was established to develop and facilitate the precise quantification of both local deck settlement and global pier inclination. Using a cross-river bridge in Hubei Province as an engineering case study, the proposed method successfully identified an average continuous settlement of 8.2 mm in the left deck area and a maximum pier offset of 182.2 mm, demonstrating its practical engineering value.

This study focused on the approach span of a specific cross-river bridge in Hubei Province as the research subject. An RTC360 3D laser scanner was utilized to collect point cloud data, and preprocessing operations—such as denoising, simplification, and sampling—were conducted using Cyclone and CloudCompare software (v2.13.2). For monitoring deck deformation, point clouds from the bottom of the transverse diaphragms and longitudinal ribs were extracted, and deflection was quantified by analyzing deviations in center point coordinates through plane fitting. In terms of pier verticality detection, multi-level cross-sections of the pier point clouds were examined, with the X- and Y-direction offsets of the center points determined via polygon fitting to assess verticality. For the overall deformation analysis, an undeformed finite element model of the bridge was created in ABAQUS and converted into point clouds. Subsequently, pre- and post-deformation point clouds were registered using the ICP algorithm, and the M3C2 algorithm was employed to compute global deformation metrics. Through technological innovation, this study addresses significant challenges in TLS-based bridge monitoring, offering novel technical solutions for structural health monitoring that hold substantial theoretical implications and engineering application value.

## 2. Theoretical Framework

### 2.1. The Basic Principle of 3D Laser Scanning Technology

Three-dimensional laser scanning is an efficient technology for acquiring three-dimensional data of real-world objects. It is capable of performing 360° omnidirectional scanning and intuitively presenting results through red, green, and blue (RGB) color mapping. A three-dimensional laser scanner comprises several components, including an emitter, a receiver, a motor system, and built-in control systems such as Charge-Coupled Device (CCD) cameras. Its operating principle is based on laser ranging, where the laser emitted by the scanner is reflected off the object’s surface and captured by the receiver. The system accurately measures the distance between the instrument and the object by calculating the time difference between the emission and reflection of the laser. Simultaneously, the receiver determines the horizontal and vertical angles of the reflected laser to obtain the three-dimensional coordinates (X, Y, Z) of the target object’s surface. These coordinates are then combined with the CCD camera data to generate raw three-dimensional laser point cloud data.

### 2.2. The Principle of Point Cloud Data Acquisition

**Figure 1:**
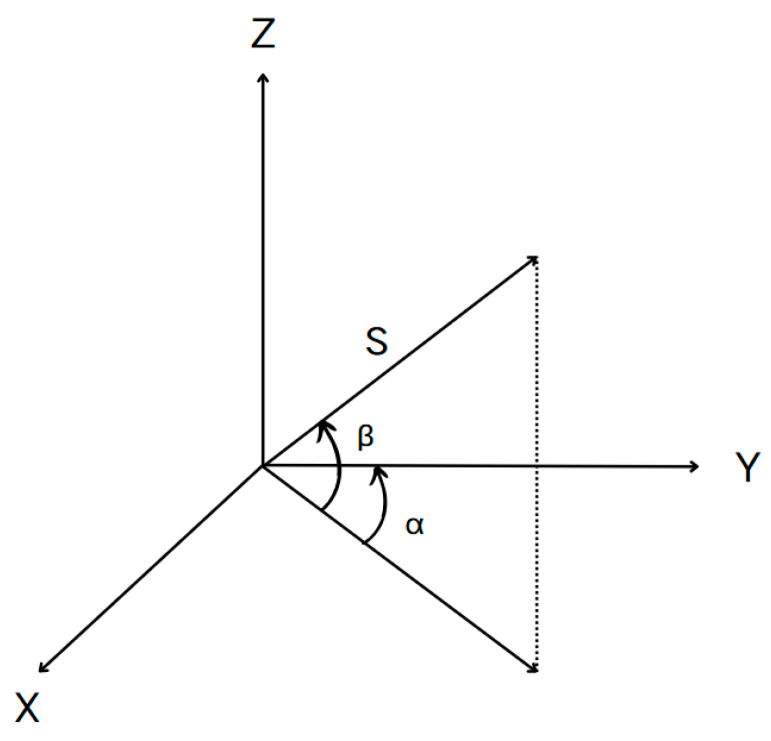
Schematic diagram of coordinate calculation for three-dimensional laser scanning.

The working principle of 3D laser scanners is based on measuring distance (range observation S) and two angles (horizontal observation α and vertical observation β) through the instrument’s integrated devices to determine the 3D coordinates of target points. The distance was calculated based on the time required for the pulse to travel from the device to the target and return [45]. The schematic diagram of the three-dimensional laser scanning coordinate calculation is shown in Figure 1.

The coordinates of the target point P can then be expressed as follows:

(1) $$\left\{\begin{array}{c} X_{i}=S\cdot \cos\beta \cdot \cos\partial \\ Y_{i}=S\cdot \cos\beta \cdot \sin\partial \\ Z_{i}=S\cdot \sin\beta \end{array}\right.$$

### 2.3. The Principle of Statistical Filtering

Statistical filtering was initially applied in the field of 2D image processing, where it was used to extract image feature textures or eliminate noise based on computed local characteristics within specified neighborhood ranges. Subsequently, its applications were extended to 3D point cloud processing, utilizing statistical analysis to identify noise points by evaluating the distances between a data point and its neighboring points. Points that did not meet the established parameter settings were classified as noise and subsequently removed.

The core principle is as follows: For each point *P_i_* in the point set, the algorithm identifies the n nearest neighboring points and calculates their average Euclidean distance to *P_i_*. The average Euclidean distance of all point clouds is assumed to follow a Gaussian distribution. The mean (μ) and standard deviation (σ) of these distances are computed to determine the characteristics of the Gaussian distribution and establish a distance threshold. If the average Euclidean distance of *P_i_* exceeds this threshold, it is considered to be noise and is removed. The specific computational steps are as follows [46,47,48,49]:

1. Calculate the average Euclidean distance between each point *P_i_* in the point set and the nearest n points, as follows:
  (2) $$d_{i}=\frac{\sum_{i=1}^{n}d_{in}}{n}$$
2. Calculate the mean value μ and the standard value σ of the average distance of all points in the point set, as follows:
  (3) $$\left\{\begin{array}{c} \mu =\frac{\sum_{i=1}^{N}d_{i}}{N}\\ \sigma =\sqrt{\frac{\sum_{i=1}^{N}{\left(d_{i}-\mu \right)}^{2}}{N-1}} \end{array}\right.$$
  where *N* is the total number of points in the point set.
3. Determine the distance threshold *d_m_*, $d_{m}=\mu +\lambda \sigma$. Compare the sizes of *d_i_* and *d_m_*. If they are greater than the threshold, eliminate the corresponding points. It is evident that this method requires the determination of two parameters to effectively remove noise: the number of neighboring points n, and the distance threshold *d_max_*. In practical applications, these parameters should be adjusted based on the specific context.

### 2.4. Point Cloud Downsampling Based on the Octree Method

Point cloud reduction based on the octree method is carried out as follows:

First, determine the overall range size of the point cloud. The boundaries of length, width, and height are determined by the difference between the maximum and minimum values of the x, y, and z coordinate axes. Secondly, represent the octree hierarchically as follows:

(4) $$d_{0}\times 2^{n}\geq d_{\max}$$

where *d_0_* is the minimum cube size and *d_max_* is the maximum cube size. Then, determine the position of the cube where the point cloud is located, as follows:

(5) $$\left\{\begin{array}{c} p=[\left(x-x_{\min}\right)/d_{0}]\\ l=[\left(y-y_{\min}\right)/d_{0}]\\ i=[\left(z-z_{\min}\right)/d_{0}] \end{array}\right.$$

where p, l, and i are index values, while x, y, and z are point cloud coordinates. Finally, the center coordinates of the minimum cube are calculated according to the formula, and the point cloud with the minimum distance is retained.

### 2.5. The Iterative Closest Point (ICP) Registration Algorithm

The Iterative Closest Point (ICP) algorithm enables the source point cloud and the target point cloud to correspond spatially by finding an optimal rigid body transformation. Given two point clouds in three-dimensional space—the source point cloud $S=\left\{s_{i}\right.{\}}_{i=1}^{N_{m}}(s_{i}\in R^{3},N_{s}\in N)$ and the target point cloud $M=\left\{m_{i}\right.{\}}_{i=1}^{N_{m}}(m_{i}\in R^{3},N_{s}\in N)$—and assuming that the rotation matrix is R and the translation vector is t, the three-dimensional point cloud registration optimization problem can be written as follows:

(6) $$\underset{R,t,c(i)}{\min}{\sum_{i=1}^{N_{S}}\left\|Rs_{i}+t-m_{c(i)}\right\|}_{2}^{2}$$

where *s_i_* is any point i in the source point cloud, and c(i) is the corresponding point of any point i in the source point cloud *S* in the target point cloud *M*. The ICP algorithm uses an iterative solution to obtain the rotation matrix ***R*** and translation vector *t*.

Given the root-mean-square error $\varepsilon_{k}={\sum_{i=1}^{N_{S}}\left\|Rs_{i}+t_{k}-m_{ck(i)}\right\|}_{2}^{2}$ of the source point cloud and the target point cloud under the rigid body transformation *R_k_* and *t_k_*, the iteration end condition holds true for the given root-mean-square $\varepsilon_{\min}$. If $\varepsilon_{k}$ ≤ $\varepsilon_{\min}$, or if the number of iterations reaches the maximum number of iterations, the algorithm terminates; otherwise, the iteration continues.

## 3. Plane Fitting Based on the Least-Squares Method

### 3.1. Plane Fitting

The Ordinary Least Squares (OLS) method is a mathematical optimization technique that aims to find the best functional fit for data by minimizing the sum of squared errors. In the context of point cloud plane fitting, this method necessitates that researchers minimize the average distance from all data points to the fitted plane, which places significant demands on the quality of the point cloud. The specific procedure is as follows:

The coordinates of any point in the point cloud region *M* are denoted as *M_i_*(x_i_, y_i_, z_i_), where i = 0, 1, 2, ..., n − 1. The general plane equation ax + by + cz + d = 0 is transformed into z = a_0_x + a_1_y + a_2_, and an objective function S is established. To minimize the average distance from *M_i_* to the fitted plane, i.e., min [S], the following conditions should be met: $\frac{\partial S}{\partial a_{k}}=0$, k = 0, 1, 2.

(7) $$S=\frac{\sum_{i=0}^{n-1}{\left(a_{0}x_{i}+a_{1}y_{i}+a_{2}-z_{i}\right)}^{2}}{n}$$

By using the above formula, a_0_, a_1_, and a_2_ can be obtained, which are the coefficients of the main plane of the point cloud *M*. The equation of the fitted plane is set as follows:

(8) ax+by+cz+d=0

The constraint condition is

(9) $$a^{2}+b^{2}+c^{2}=1$$

The parameters of the plane, denoted as a, b, c, and d, can then be determined. To ensure that the resulting fitted plane is optimal, it is essential to minimize the sum of the squares of the distances from the points to this plane, thereby satisfying the following conditions:

(10) $$e=\sum_{i=1}^{n}d_{i}\to \min$$

where d_i_ is the distance from any point pi (xi,yi,zi) in the point cloud M to this plane, and $d_{i}=\left|a_{i}\right.+b_{i}+c_{i}+\left.d\right|$. To ensure that e→min, it can be obtained through matrix decomposition using Singular Value Decomposition (SVD).

### 3.2. Extraction of the Center Point of the Point Cloud Slice Fitting Plane

According to the formula for calculating the distance from a point to a straight line, if this concept is further extended to determine the distance from a point to a plane, the following formula can be derived:

(11) $$d_{i}=\frac{\left|\alpha^{\prime }x_{i}+\beta^{\prime }y_{i}+\gamma^{\prime }z_{i}\right|}{\sqrt{\alpha^{\prime 2}+\beta^{\prime 2}+\gamma^{\prime 2}}}$$

In the above formula, $\alpha^{\prime },\;\beta^{\prime },\;\gamma^{\prime }$ are the reference plane parameters.

The distance can be obtained by using the above formula, i.e., the distance from any point at one end of the point cloud to the reference plane. At this point, by setting the distance from the first required slice point cloud to this reference plane, the three-dimensional coordinate data of the initial slice point cloud can be obtained, which is denoted as $\left(x_{i}^{1},y_{i}^{1},z_{i}^{1}\right)$; then, the coordinates of the center point of the initial slice point cloud can be expressed as follows:

(12) $$\left\{\begin{array}{c} \overset{—}{x}=\frac{1}{m}\sum_{i=1}^{m}x_{i}^{1}\\ \overset{—}{y}=\frac{1}{m}\sum_{i=1}^{m}y_{i}^{1}\\ \overset{—}{z}=\frac{1}{m}\sum_{i=1}^{m}z_{i}^{1} \end{array}\right.$$

where m represents the total number of points contained in the initial slice point cloud.

If the three-dimensional coordinate data of the initial slice point cloud and the calculation method of the center point coordinates are extended to the subsequent n slice point clouds obtained, then the three-dimensional coordinate data of the n-th point cloud slice can be recorded as $\left(x_{i}^{n},y_{i}^{n},z_{i}^{n}\right)$; therefore, the expression for the coordinates of the center point is as follows:

(13) $$\left\{\begin{array}{c} \overset{—}{x}=\frac{1}{m}\sum_{i=1}^{m}x_{i}^{n}\\ \overset{—}{y}=\frac{1}{m}\sum_{i=1}^{m}y_{i}^{n}\\ \overset{—}{z}=\frac{1}{m}\sum_{i=1}^{m}z_{i}^{n} \end{array}\right.$$

## 4. The Principle of the M3C2 Algorithm

**Figure 2:**
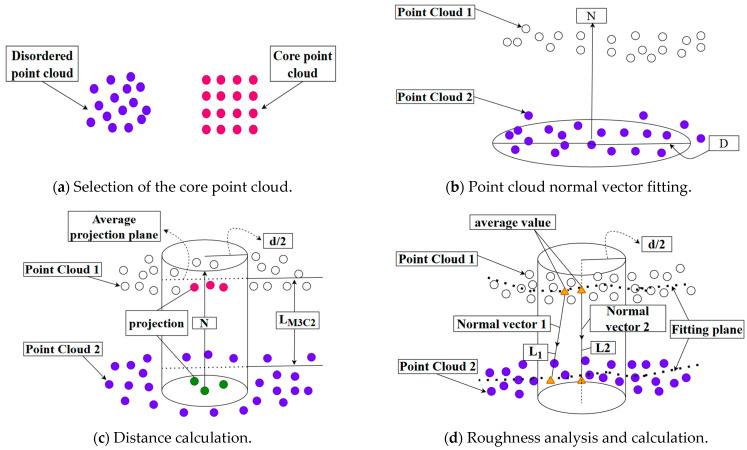
The principles of the M3C2 algorithm.

For the comparative analysis of 3D point cloud models from different time periods, the M3C2 point cloud comparison algorithm was utilized. This procedure involves selecting core point clouds, calculating 3D surface normals, computing distances between point clouds, and determining confidence intervals for spatial variables, as illustrated in Figure 2. This algorithm facilitates the direct detection of complex terrain changes in point clouds, without the need for meshing. Furthermore, during the change computation, it exhibits minimal sensitivity to spatial point density, surface roughness, and variations in sampling positions [50].

Because selecting an appropriate core point cloud can significantly improve computational efficiency, in the initial stage, the original data must be downsampled according to the set distance to obtain a core point cloud with a lower density and uniform distribution and use it as the basis for change recognition, as shown in Figure 2a. After selecting the appropriate core point cloud, for any given core point *P_core_* within a radius of D/2, a plane can be fitted with other point cloud data in the neighborhood. From this, the local normal vectors N of the two point clouds are obtained, as shown in Figure 2b, and the core point *P* is noted. The distances from all points within the radius D/2 to the optimal fitting plane are characterized by roughness σ(D), as follows:

(14) $$\sigma =\sqrt{\frac{\sum_{k=1}^{M}{\left(a_{k}-\bar{a}\right)}^{2}}{M}}$$

where $a_{k}$ is the distance between the k-th point within the radius D/2 and the best-fitting plane, $\bar{a}$ is the average distance between the best-fitting plane and all point clouds within the range of D/2, and *M* represents the total amount of point clouds distributed within the radius range of D/2.

Starting from the fitting plane, where *P_core_* is located along the normal direction with d/2 as the projection radius, there exists a cylinder passing through *P_core_* with the normal vector *N* as the axis and intersecting the point clouds of the two periods. A search is conducted for all point clouds n_1_ and n_2_ contained in the cylinder plane, and the average positions of all point clouds n_1_ and n_2_ contained in the two period point cloud columns, respectively, along the normal vector, are calculated. At this time, the average positions of the two period point clouds in the columns are *M_1_* and *M_2_*, respectively, and the difference between the two average positions is the distance *L_M3C2_*.

## 5. Applied Research on Bridge Deformation Detection

**Figure 3:**
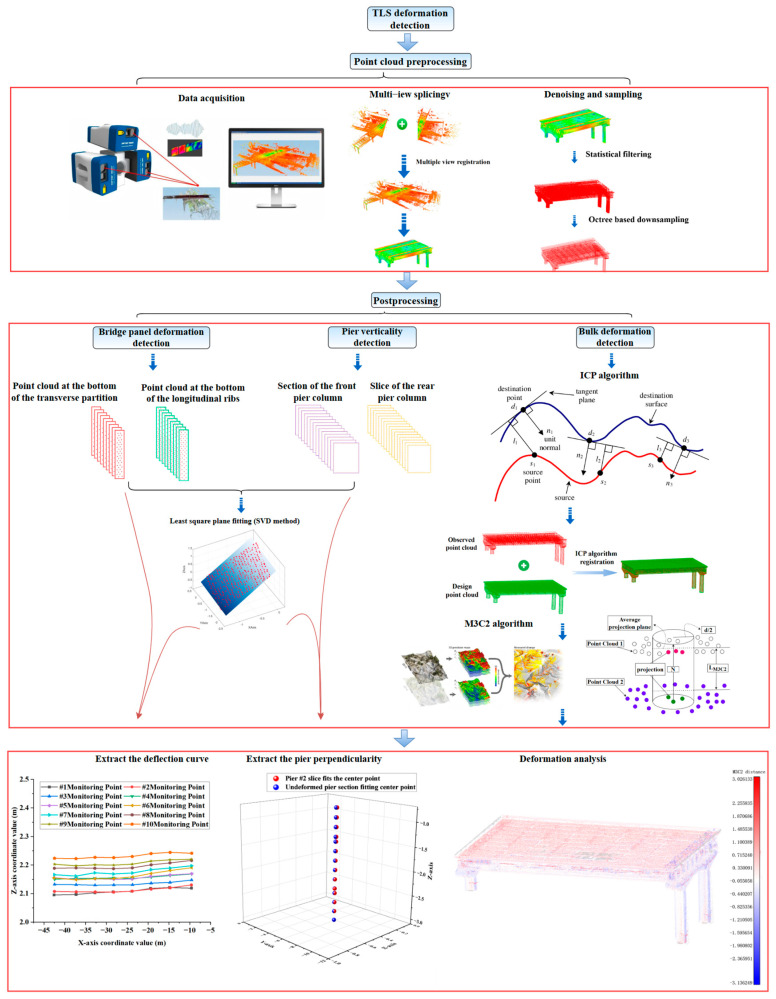
Technical flowchart.

Building upon existing research, this paper proposes a method for analyzing bridge deformation based on least-squares polygon plane fitting of feature point clouds. This study focuses on the approach span section of a specific cross-river bridge in Hubei Province. An RTC360 3D laser scanner was utilized to capture real-time 3D point cloud data of the bridge’s overall deformation. The acquired point cloud data underwent preprocessing, which included denoising, registration, and simplification, using Open3D algorithm programs and CloudCompare software. The technical roadmap is illustrated in Figure 3.

### 5.1. Data Collection

**Figure 4:**
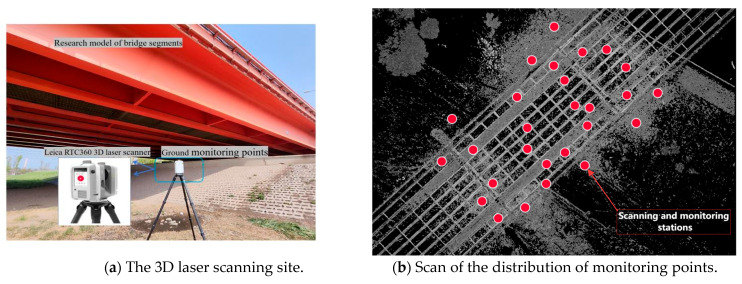
On-site observation of real scenes by 3D laser scanner.

The specific cross-river bridge in Hubei Province, located in Wuhan City, is a “hybrid-type” cable-stayed bridge that features steel box girders in the central section of its main span and concrete box girders at both ends. The girder structure utilizes a bolted–welded hybrid configuration, with the steel box girder measuring a total length of 906.4 m. This study focuses on the approach span section of the bridge, which is approximately 42 m long and 27 m wide. The segment of the bridge under investigation consists of front piers located onshore and rear piers that are partially submerged underwater, as illustrated in Figure 4.

To address the deformation monitoring requirements of the approach span of a certain cross-river bridge in Hubei Province, this study employed the Leica RTC360 3D laser scanner for data acquisition (the technical parameters are listed in Table 1). To minimize the impacts of environmental interference and vehicle vibrations on monitoring accuracy, the 3D laser scanning survey commenced at 3 p.m. on March 28, 2024, under optimal environmental conditions: temperature of 23 °C and south wind speed ≤ Level 3, both meeting the standard requirements for precise scanning measurements. Although environmental noise during data collection is unavoidable, the Leica RTC360 3D laser scanner utilizes a multichannel receiver that actively enhances signal integrity. This system effectively separates target signals from ambient noise in real time while directly suppressing high-frequency noise through hardware-level filter circuits, thereby improving the signal-to-noise ratio (SNR) of the original signal. To achieve dual-stage noise reduction, additional filtering was applied during point cloud preprocessing using CloudCompare software.

The device integrates multiple innovative technologies: (1) a high-efficiency scanning capability (2 million points/second), achieving a 3-fold improvement compared to conventional equipment; (2) automatic point cloud registration based on a Visual Inertial System (VIS), eliminating the need for targets or common points while reducing manual intervention by 80%; and (3) high-precision data acquisition (with a ranging accuracy of ±1 mm + 10 ppm) combined with noise suppression algorithms, enabling a feature resolution of up to 0.5 mm (e.g., for identification). These characteristics ensure the reliability and detailed integrity of high-density point cloud data, establishing a precise foundation for deformation analysis.

**Figure 5:**
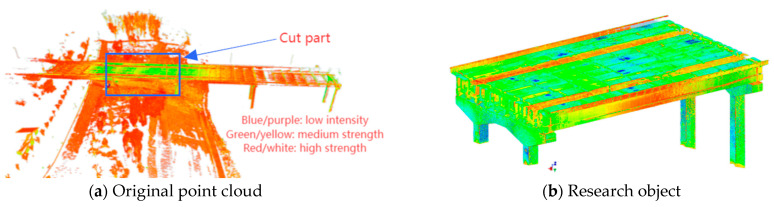
Research object of deformation detection.

The approach bridge section was scanned at multiple locations using the Leica RTC360 3D laser scanner. The original point cloud of the bridge, after splicing, is presented in Figure 5a. Since the accuracy of 3D laser scanning is influenced by spatial distance and environmental factors, the original point cloud was partially simplified to retain key features and enhance the data processing efficiency. Ultimately, the point cloud model of the box girder bridge segment was extracted as the subject for deformation monitoring, as illustrated in Figure 5b. The constraint ID is 39, the splicing station is 27, the weight factor is 1, and the error vector remains within the specified tolerance threshold. The overall splicing accuracy of the bridge meets the monitoring requirements.

### 5.2. Data Processing

The raw point cloud data collected from field surveys typically contain noise, redundancy, and unregistered discrete points, rendering them unsuitable for direct model construction. Consequently, preprocessing the point cloud data through office processing workflows is necessary, primarily involving the following key steps:

1. Point Cloud Denoising: Statistical Outlier Removal (SOR) and Weighted Principal Component Analysis (WPCA) algorithms can be employed to remove unstructured noise caused by environmental interference or equipment errors.
2. Point Cloud Simplification: Optimized algorithms based on octree downsampling can be employed to reduce data redundancy while preserving essential features.
3. Point Cloud Registration: Enhanced 4PCS or Iterative Closest Point (ICP) algorithms can be utilized to align multi-temporal or multi-station point clouds into a unified coordinate system.

#### 5.2.1. Point Cloud Noise Reduction

During the application of 3D laser scanning technology, the data acquisition phase is vulnerable to interference from various sources. These include, but are not limited to, the inherent limitations of the equipment, dynamic environmental factors (such as moving vehicles, pedestrians, and the sway of vegetation within the scanning area), airborne particulate matter, and geometric edge effects. Such sources of interference introduce non-structural noise points (e.g., outliers and drifting points) into the point cloud data, significantly undermining the accuracy of subsequent modeling and deformation analysis. Therefore, in practical operational workflows, it is essential to implement preliminary manual screening in conjunction with appropriate filtering algorithms to eliminate these non-target redundant data. This approach enhances data quality and improves the reliability of the analytical results.

**Figure 6:**
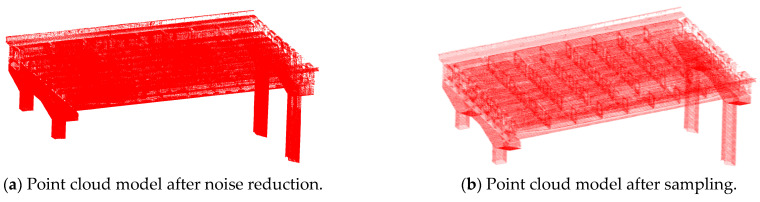
Noise reduction and point cloud model after sampling.

Given the irregular shape of the point cloud model, this study employed the Statistical Outlier Removal (SOR) method for denoising. The number of neighboring points used to calculate the average distance was set to six, and the threshold was determined based on the standard deviation of the average distance within the point cloud, with the threshold value established at one. This method utilizes local neighborhood statistical characteristics to identify and eliminate noise points (e.g., outliers) that deviate from statistical norms by analyzing the distance distribution between a target point and its neighboring points. As a classic point cloud denoising algorithm, the SOR method offers broad applicability, strong robustness, and high precision, making it a reliable technical solution for denoising irregular point cloud models. The denoised point cloud, as illustrated in Figure 6a, demonstrates effective filtering while preserving the model’s complete geometric shape. The statistical filtering algorithm employed for point cloud denoising requires manual input of the number of neighborhoods and distance threshold coefficients in the initial stage. In the subsequent stage, the algorithm program is automatically executed to process the point cloud data, with a total running time of three minutes.

#### 5.2.2. Point Cloud Sampling

Due to the substantial volume of raw point cloud data (initial point count: 79,540,078 points), significant system resources are consumed during storage, processing, and visualization, resulting in a marked reduction in computational efficiency (with typical processing times increasing by 50% to 70%). Consequently, point cloud simplification is necessary to balance the data volume with feature preservation. This study employed an octree-based downsampling method, implemented in CloudCompare, with the subdivision level set to 8 (higher levels correspond to smaller voxels and a greater number of retained points). By recursively partitioning spatial grids and retaining representative points within each grid (such as centroids or points of highest density), effective point cloud compression was achieved. As illustrated in Figure 6b, the processed point count was reduced from 79,540,078 to 1,969,086 points, representing a 97.52% reduction. The experimental results demonstrate that this method significantly decreases redundant data and computation time while effectively preserving critical model features (with a feature retention rate of ≥95%). This provides an efficient and reliable data foundation for subsequent analyses. The downsampling method based on the octree structure requires manual input of the octree level in the initial stage, after which the algorithm is executed to simplify the point cloud data. The running time of the algorithm is 2 min.

#### 5.2.3. Point Cloud Registration

The point cloud registration process can be divided into two stages: coarse registration and fine registration. Coarse registration involves aligning point clouds when their relative poses are entirely unknown. This stage seeks to find a rotation–translation transformation matrix that brings the two point clouds into approximate alignment, thereby transforming the target point cloud data into a unified coordinate system and providing a suitable initial value for fine registration. Fine registration, on the other hand, focuses on minimizing the spatial position differences between point clouds based on the results of coarse registration to obtain a more accurate rotation–translation transformation matrix.

**Figure 7:**
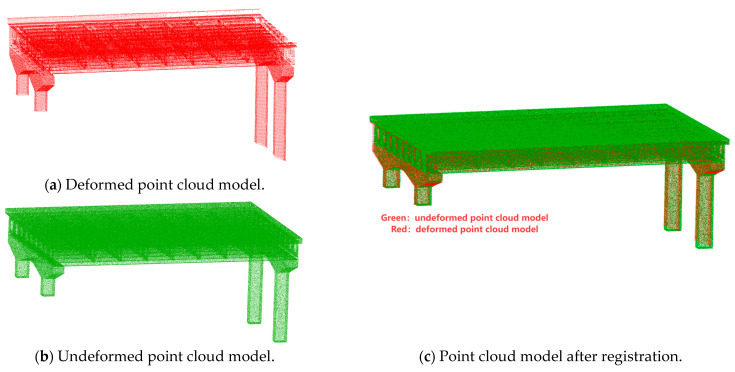
ICP registration.

This study employed a two-stage registration method, which includes coarse registration followed by ICP-based fine registration. During the registration process, manual placement of markers or target points is unnecessary; instead, the bridge piers, which exhibit minimal geometric deformation and possess easily identifiable artificial features, serve as registration markers. It is important to note that, prior to executing ICP registration, the original point cloud data must be downsampled. The ICP registration is applied to both the pre-deformation design point cloud model and the post-deformation measured point cloud model after denoising and sampling. The registered point cloud is illustrated in Figure 7.

### 5.3. Overall Deformation Detection of Bridges

After completing the preprocessing of the point cloud, deformation detection is performed through multi-scale registration and comparison. First, the point clouds representing the state before and after deformation are downsampled using the octree method to create a core point cloud with uniform density. Next, coarse registration and the ICP algorithm are employed to achieve fine registration, aligning the measured point cloud with the designed point cloud within the same coordinate system. To quantify the overall deformation, the M3C2 algorithm is utilized. The efficiency of neighborhood searches is enhanced through the use of the octree, and the displacement distribution is visualized using chromaticity mapping.

**Figure 8:**
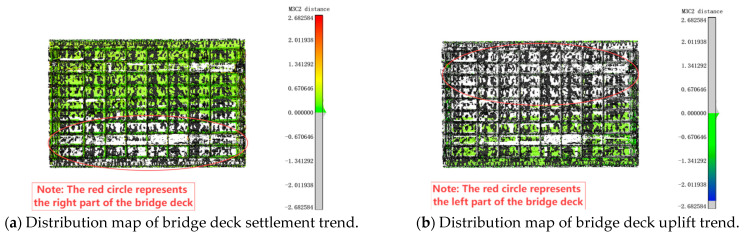
Identification results of M3C2 algorithm.

As illustrated in Figure 8, the displacement distribution is represented using red, green, and blue colors according to the reference color scale. The green areas correspond to the smallest displacement values and are the most widely distributed, indicating minimal overall deformation of the bridge. The sign (positive or negative) of the displacement is determined by the direction of the fitted plane’s normal vector, *N*, at each point in the compared point cloud. As shown in Figure 2c, if the equivalent point in the reference point cloud lies on the side indicated by the normal vector *N*, the M3C2 distance is positive; if it lies on the opposite side, the distance is negative. During the initial stage of overall deformation detection of the bridge, the core distance and projection radius of the M3C2 algorithm must be manually set. Subsequently, the distance field between the point clouds is automatically calculated using the startup algorithm program. The algorithm’s running time is 3 min.

Further analysis of the deck displacement distribution reveals significant differences in deformation trends between the two sides of the bridge. Using the central axis of the deck as the dividing line, Figure 8a illustrates that a substantial area on the left side of the bridge exhibits positive displacement values, indicating settlement in comparison to the undeformed deck. Conversely, Figure 8b shows that the right side of the bridge displays negative displacement values, suggesting a degree of uplift relative to the left side. Additionally, the bridge piers exhibit noticeable offset phenomena.

### 5.4. Bridge Deck Deflection Monitoring

Deflection monitoring is a critical indicator for assessing the structural health of bridges. Deflection refers to the displacement of points along the axis of a beam or column within its normal plane when deformation occurs under load. In the context of bridges, deck deflection monitoring primarily focuses on two directions: (1) vertical deflection, which is the vertical displacement along the deck axis (Z-direction); and (2) lateral deflection, which is the horizontal displacement perpendicular to the deck axis (X/Y-direction). As the primary load-bearing component for traffic loads on bridges, deck deformation is predominantly induced by bending effects, and variations in its deflection directly reflect the overall stiffness and load-bearing capacity of the bridge.

**Figure 9:**
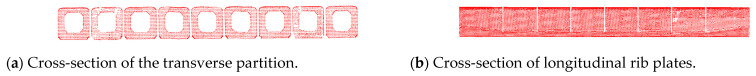
Cross-sections of the transverse partition and longitudinal ribs.

**Figure 10:**
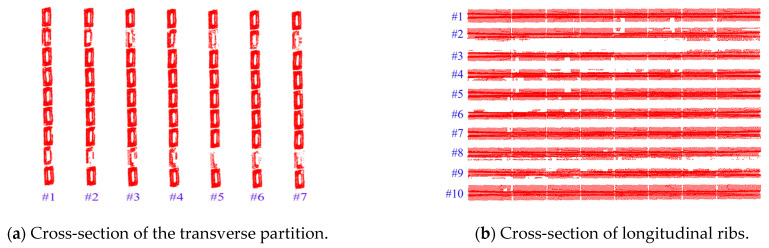
The inspection object numbers of the transverse partition and longitudinal ribs.

**Figure 11:**
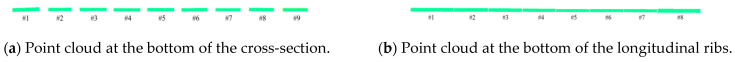
Point cloud extraction from the bottom of the transverse partition and the longitudinal rib plate.

For the measured point cloud model in this study, the bottom point clouds of the transverse partitions and the longitudinal ribs were selected as the monitoring objects. The center point coordinates of the feature point clouds were extracted using the least-squares plane-fitting method and designated as the deflection monitoring points. The specific methods are outlined as follows:

1. Diaphragm Deflection Curve Extraction: Seven diaphragm cross-sections were selected, and nine monitoring points were extracted from the bottom point cloud of each diaphragm. The variation in vertical displacement (Z-direction) at the monitoring points within the X-Z plane was analyzed. The cross-sectional schematic diagram of the transverse partition is shown in Figure 9a, the cross-sections of each selected transverse partition are shown in Figure 10a, and the point cloud at the bottom of the transverse partition is shown in Figure 11a.
2. Longitudinal Rib Deflection Curve Extraction: Ten longitudinal rib cross-sections were selected, and eight monitoring points were extracted from the bottom point cloud of each rib. The variation in vertical displacement (Z-direction) of the monitoring points in the Y-Z plane was analyzed. The schematic diagram of the longitudinal rib section is shown in Figure 9a, the selected longitudinal rib sections are shown in Figure 10a, and the point cloud at the bottom of the longitudinal ribs is shown in Figure 11a.

**Figure 12:**
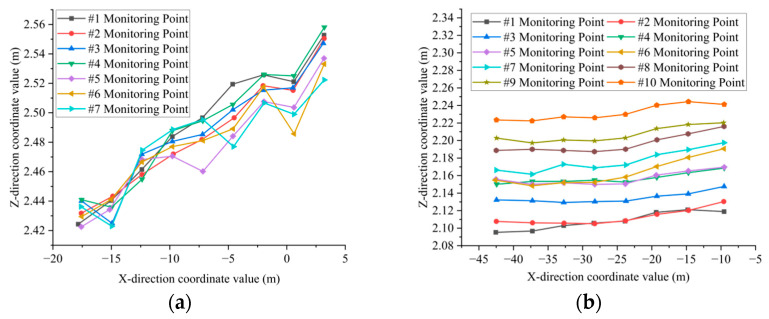
Coordinate values of the center points of the monitoring points: (**a**) The coordinate values of the center points of monitoring points #1 to #7 at the bottom of the transverse partition. (**b**) The coordinate values of the center points of monitoring points #1 to #7 at the bottom point cloud of the longitudinal rib.

**Figure 13:**
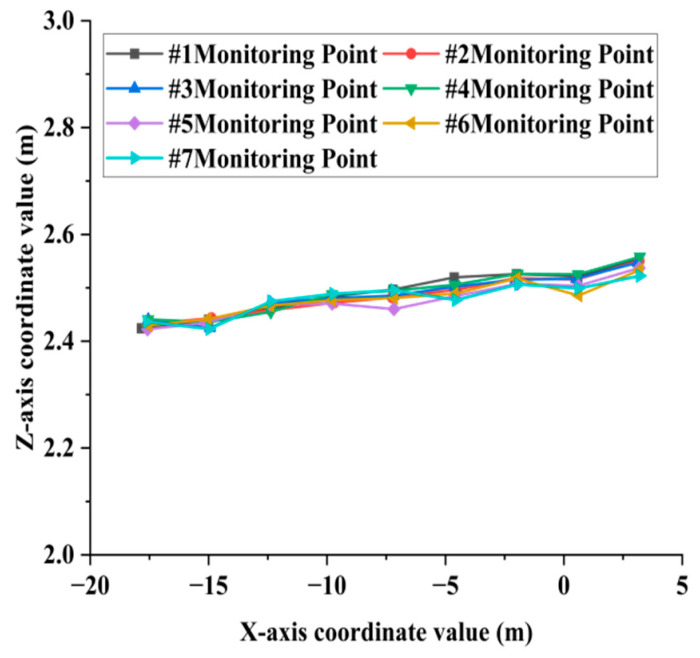
Deflection curve of the transverse partition.

**Figure 14:**
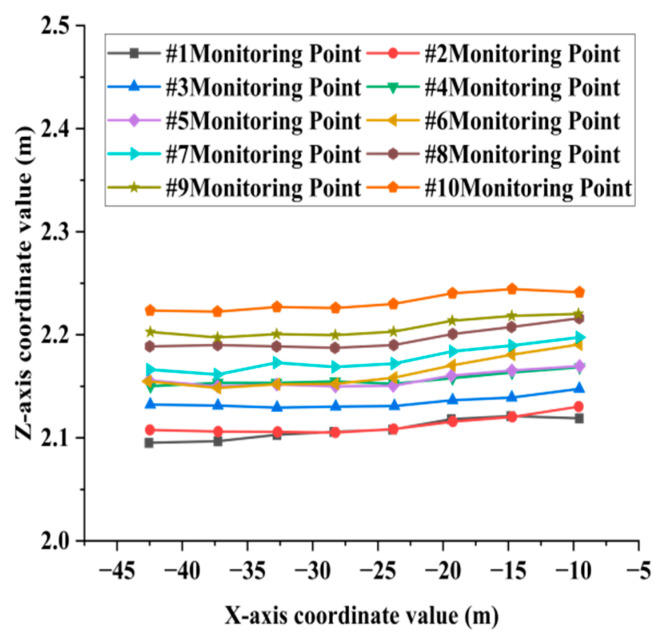
Deflection curve of longitudinal rib plates.

When extracting the deflection curve of the bridge deck, it is essential to manually extract the bottom feature point clouds of the transverse partitions and longitudinal ribs as monitoring points. Subsequently, planar fitting of the feature point cloud is performed, and the coordinates of the fitting center points are automatically calculated using the algorithm. The algorithm has a running time of 7 min. The coordinate values of the center points of the monitoring points of the transverse partition and the longitudinal ribs in the X-Z direction are shown in Figure 12a,b. The deflection distribution characteristics of the diaphragms and longitudinal ribs are illustrated in Figure 13 and Figure 14, respectively.

#### Analysis of Bridge Deck Deflection Monitoring Results

As illustrated in Figure 13, according to the deflection curves of the transverse partition #1 to #7, it can be seen that the elevation coordinates of all monitoring points exhibit an overall upward trend in the direction perpendicular to the central axis of the bridge deck. Specifically, the elevation on the left side of the bridge deck is significantly lower than that on the right side. This deformation pattern aligns with the current traffic conditions. The primary route for heavy trucks crossing the bridge is from Wuchang to Hanyang. Consequently, the traffic load on the left side of the approach bridge deck is greater than that on the right side.

Figure 14 illustrates the deflection curves of longitudinal rib plates #1-#10, demonstrating a consistent downward trend in elevation coordinates for all monitoring points along the central axis of the bridge deck. This indicates uniform subsidence of the deck in the longitudinal direction. The observed trend in settlement deformation can be attributed to the long-term erosion of the bridge foundation, situated in the lower section of the riverbed, caused by water flow. Consequently, the variation in the extracted deformation curve aligns with the geological conditions of the bridge’s location.

### 5.5. Bridge Pier Verticality Monitoring Based on Point Cloud Continuous Slice Extraction Technology

The vertical alignment of bridge piers is a critical indicator for assessing the quality of bridge pier column construction. When subjected to external loads, the bridge piers must be capable of withstanding the force conditions imposed by the bridge load. According to the “General Code for Design of Highway Bridges and Culverts”, the permissible deviation in the vertical alignment of bridge piers and columns is 0.3%, with a maximum allowable deviation of 20 mm.

**Figure 15:**
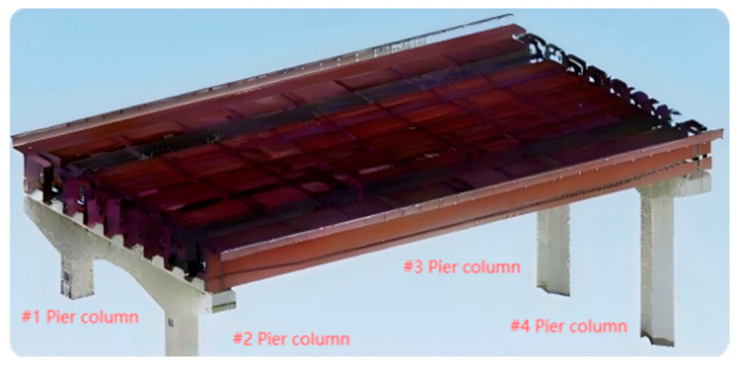
Bridge pier column numbers.

**Figure 16:**
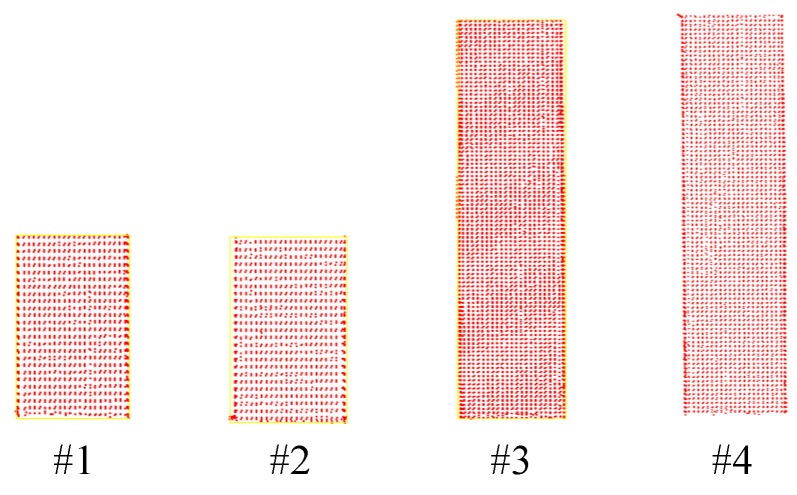
Bridge pier point clouds #1–#4.

**Figure 17:**
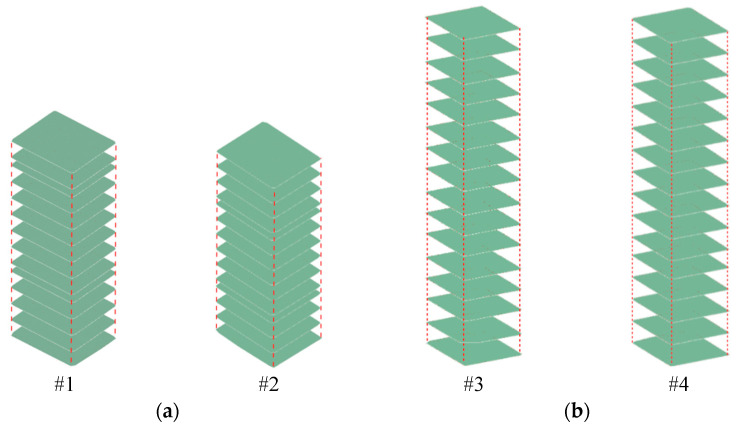
Continuous point cloud slices of pier columns #1 to #4: (**a**) Schematic diagram of point cloud slices from #1 to #2 of the front pier columns. (**b**) Schematic diagram of point cloud slices from #3 to #4 of the rear pier columns.

In this paper, front pier columns #1 and #2, as well as rear pier columns #3 and #4, were selected as the objects for deformation monitoring, as illustrated in Figure 15 and Figure 16. Following the pruning and noise reduction of the bridge pier point cloud using the Open3D point cloud library, a multi-level slicing method was employed to extract geometric features. Front piers #1 and #2 were sliced with a thickness of 0.05 m and an interval of 0.13 m, resulting in 14 slices extracted from each. In contrast, back piers #3 and #4 were sliced at a thickness of 0.125 m and an interval of 0.45 m, yielding 16 slices from each, as depicted in Figure 17. The geometric centers of each slice were determined through polygonal plane fitting, and the center axes of the bridge piers were fitted using the least-squares method to calculate their verticality. When assessing the verticality of bridge piers, the point cloud of the pier column is initially processed manually using a method of continuous and equidistant slicing. Subsequently, plane fitting is performed on the slices, and the coordinates of the fitting center point are automatically calculated with the assistance of an algorithm. The total execution time of the algorithm is 6 min.

The verticality (⟂) calculation takes the center of the pier’s bottom section as the reference, analyzes the coordinate deviation (∆D) between the top of the pier and the center of the pier bottom and its height difference (H), and quantifies the degree of inclination through the slope formula. The calculation formula is as follows:

(15) $$⊥=\frac{\Delta D}{\Delta H}$$

According to the “Code for Acceptance of Construction Quality of Concrete Structure Engineering” (50204-2014), the allowable verticality is denoted by [⟂], and its calculation formula is as follows:

(16) $$[⊥]=\frac{\Delta D}{H}\leq \frac{1}{1000}$$

#### Analysis of the Verticality Detection Results of Pier Columns

**Figure 18:**
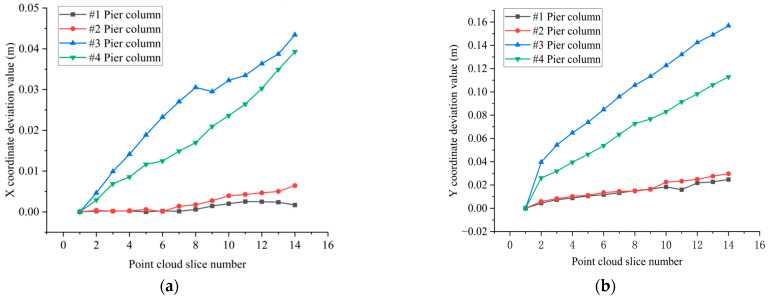
The deformation of the slice center points of piers #1 to #4 in the X- and Y-directions: (**a**) The deviation of the displacement of the center point of the cloud slice of piers #1 to #4 in the X-direction. (**b**) The deviation of the displacement of the center point of the cloud slice of piers #1 to #4 in the Y-direction.

**Figure 19:**
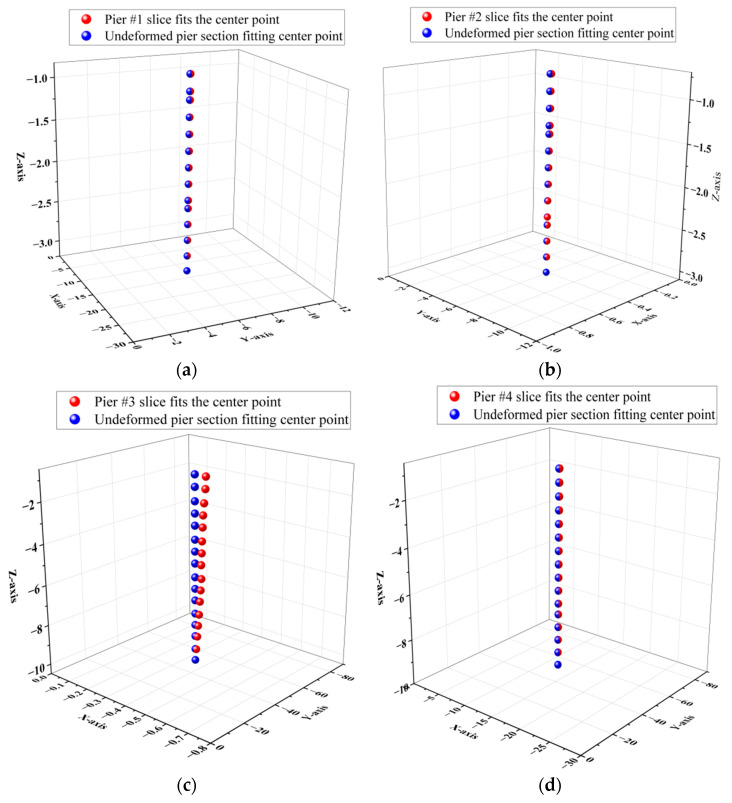
Three-dimensional scatter plots of the fitting centers of piers #1 to #4 sections: (**a**) Comparison of the center points of the continuous slices of pier #1 before and after deformation. (**b**) Comparison of the center points of the continuous slices of pier #2 before and after deformation. (**c**) Comparison of the center points of the continuous slices of pier #3 before and after deformation. (**d**) Comparison of the center points of the continuous slices of pier #4 before and after deformation.

As illustrated in Figure 18, the increase in the number of point cloud slices along the elevation direction correlates with an upward trend in the deformations of the slice center point coordinates for piers #1 to #4 in both the X- and Y-directions, consistent with the established deformation patterns of piers. Notably, the deformation of the slice center point in the Y-direction exceeds that in the X-direction. The deformations of the rear piers #3 and #4 in the X-direction remain within 60 mm, while the Y-direction deformations are contained within 180 mm. In contrast, the deformations of the front pier columns #1 and #2 in the X-direction are limited to 7 mm, and those in the Y-direction are restricted to 30 mm. Furthermore, as shown in Figure 19 and Table 2, the inclination angle, offset, and verticality of the rear pier columns #3 and #4 are significantly greater than those of the front pier columns #1 and #2. This indicates that the offset deformation of the front pier columns is less pronounced than that of the rear pier columns.

## 6. Discussion

This study successfully achieved a refined analysis of bridge structural deformation by integrating 3D laser scanning technology with multi-scale algorithms. The M3C2 algorithm demonstrated significant advantages in global deformation detection, enabling the direct comparison of multi-temporal point clouds without the need for meshing. This effectively overcomes the limitations of traditional methods (e.g., DOD, C2C) in handling complex geometric features. The results revealed that the settlement on the left side of the deck averaged 8.2 mm, while the pronounced inclination of the rear pier reached a maximum offset of 182.2 mm. These measurements were closely correlated with the transverse distribution of traffic loads and geological conditions, thereby validating the dynamic relationship between bridge deformation and external loads or environmental factors. These findings align with the conclusions of Lichti et al. [40] and Zogg et al. [41] regarding the accuracy of TLS in monitoring bridge deformation. However, this study further enhanced the precision of local feature analysis by introducing multi-scale algorithms and plane-fitting techniques.

Notably, the deformation of the rear pier column is more pronounced than that of the front pier column. This discrepancy can be attributed to the scouring and erosion of the foundation of the rear pier column due to river flow, which aligns with the deformation distribution pattern reported by Xu et al. [44] in their landslide monitoring study. Furthermore, the verticality detection method based on point cloud slicing and plane fitting offers a more comprehensive representation of the spatial deformation patterns of the piers compared to traditional single-point measurements, providing precise quantitative support for pile foundation reinforcement projects. The statistics of deformation errors in three directions are presented in Table 3. The monitoring error for the bridge deck deflection was ±1.2 mm, while the monitoring error for the verticality of the bridge piers was ±2.8 mm, both of which significantly exceed the JTG/AASHTO standard. Owerko et al. [50] achieved the high-precision standards required for steel bar detection through the integration of ground laser scanning technology and computer technology, confirming that the accuracy of TLS is ≤±4 mm, which is consistent with the accuracy observed in the X-, Y-, and Z-directions, as shown in Table 3.

Nevertheless, this study has certain limitations. First, the monitoring range of 3D laser scanning is limited; the RTC360′s maximum effective distance is 130 m, necessitating multi-station scanning for large-span bridges, which may introduce registration errors. Second, the parameter settings for statistical filtering during point cloud denoising and simplification are based on empirical knowledge, which may affect the completeness of feature retention. Finally, while the M3C2 algorithm mitigates the impact of point cloud density and surface roughness, the computational efficiency for processing high-density point clouds requires further optimization to meet real-time monitoring demands. The multi-scale analysis method proposed in this paper, which combines the M3C2 algorithm with least-squares plane fitting, can facilitate hourly monitoring of bridges and daily routine monitoring during key events such as floods. Future work will incorporate synchronized total station monitoring during bridge maintenance closures.

## 7. Conclusions

This study developed a multi-scale deformation monitoring framework that integrates TLS with advanced point cloud processing algorithms. Through a comprehensive analysis of the approach bridge span of a specific cross-river bridge in Hubei Province, several important conclusions were drawn, which hold significant implications for the advancement of structural health monitoring.

Based on the M3C2 algorithm and the least-squares plane-fitting method, it was observed that the bridge deck exhibited a deflection trend characterized by left-side settlement and right-side uplift in a direction perpendicular to the bridge axis. In contrast, the longitudinal ribs displayed overall settlement along the bridge axis. The transverse diaphragms demonstrated a general upward trend in the vertical direction, attributed to greater vehicle loads on the left side, thereby confirming the accuracy and applicability of the method. When the point cloud density of the M3C2 algorithm exceeds 500 points per square meter, the spatial resolution reaches 0.5 mm, enabling sub-millimeter-level full-field analysis suitable for complex deformation scenarios. The detection error for micro-settlement of the bridge deck was ±1.2 mm, which meets the precision requirements outlined in the “Highway Bridge Maintenance Code” (JTG H11-2004) and the standards set by the American Association of State Highway and Transportation Officials (AASHTO).

The deformation of the rear piers (#3 and #4) in both the X- and Y-directions was significantly greater than that of the front piers (#1 and #2), reaching up to 180 mm in the Y-direction. This discrepancy is primarily due to the rear piers’ location in the riverbed area, where they have been subjected to long-term scouring and erosion from water flow, resulting in excessive verticality (greater than 0.1%). Therefore, priority reinforcement is necessary. Furthermore, the permissible error for bridge pier inclination offset was maintained within ±2.8 mm, underscoring the stringent precision requirements for structural stability. 

Bridge deformation is primarily caused by uneven load distributions and variations in geological conditions. The settlement of the bridge deck occurs due to the scouring of the foundation in the underwater section, while the upward deflection of the transverse diaphragms results from heavier loads on the left side. To mitigate further deformation, it is recommended to implement pile foundation reinforcement and riverbed protection measures for the rear piers.

## Figures and Tables

**Table 1:** Technical parameters of the RTC360 3D laser scanner.

Scanning Range	0.5–130 m
Scanning rate	Up to 2,000,000 points per second
Field-of-view angle	Level: 360° Vertical: 300°
Resolution	3 mm@10 m
	6 mm@10 m
	12 mm@10 m
Built-in camera	36 million pixels
Range noise	0.4 mm@10 m, 0.5 mm@20 m
Working temperature	−5–40 °C

**Table 2:** Test results of the verticality of bridge piers.

Pier Column Number	Inclination Angle (°)	Offset (mm)	Verticality
#1	0.6103°	24.8	0.010682
#2	0.7499°	30.4	0.013078
#3	1.1350°	182.2	0.019765
#4	0.9050°	136.6	0.015813

**Table 3:** Statistics of deformation errors in three directions.

Monitoring Location	Error in the X-Direction (mm)	Error in the Y-Direction (mm)	Error in the Z-Direction (mm)
Left bridge deck	±0.8	±0.5	±1.2
Right bridge deck	±0.7	±0.4	±1.0
Front bridge pier #1	±1.0	±1.5	±0.5
Front bridge pier #2	±1.2	±1.8	±0.6
Rear bridge pier #3	±1.5	±2.8	±0.9
Rear bridge pier #4	±1.3	±2.5	±0.8

## Data Availability

Data will be made available upon request.
